# Secondary Prevention of Cervical Cancer: ASCO Resource–Stratified Guideline Update

**DOI:** 10.1200/GO.22.00217

**Published:** 2022-09-26

**Authors:** Surendra S. Shastri, Sarah Temin, Maribel Almonte, Partha Basu, Nicole G. Campos, Patty E. Gravitt, Vandana Gupta, Dorothy C. Lombe, Rául Murillo, Carolyn Nakisige, Gina Ogilvie, Leeya F. Pinder, Usha R. Poli, Youlin Qiao, Yin Ling Woo, Jose Jeronimo

**Affiliations:** 1 The University of Texas, MD Anderson Cancer Center, Houston, TX; 2 American Society of Clinical Oncology, Alexandria, VA; 3 IARC, Lyon, France; 4 Harvard University T.H. Chan School of Public Health, Boston, MA; 5 National Cancer Institute, Bethesda, MD; 6 V Care Foundation, Mumbai, India; 7 Regional Cancer Treatment Services, MidCentral District Health Board, Palmerston North, New Zealand; 8 Centro Javeriano de Oncología, Bogota, Colombia; 9 Mulago Hospital, Kampala, Uganda; 10 BC Women's Hospital, Vancouver, BC, Canada; 11 University of Washington, Seattle, WA; 12 India Institute of Public Health, Hyderabad, India; 13 Chinese Academy of Medical Sciences and Peking Union Medical College, Beijing, China; 14 University of Malaya, Kuala Lumpur, Malaysia

## Abstract

To update resource-stratified, evidence-based recommendations on secondary prevention of cervical cancer globally.

**METHODS:**

American Society of Clinical Oncology convened a multidisciplinary, multinational Expert Panel to produce recommendations reflecting four resource-tiered settings. A review of existing guidelines, formal consensus-based process, and modified ADAPTE process to adapt existing guidelines was conducted. Other experts participated in formal consensus.

**RESULTS:**

This guideline update reflects changes in evidence since the previous update. Five existing guidelines were identified and reviewed, and adapted recommendations form the evidence base. Cost-effectiveness analyses provided indirect evidence to inform consensus, which resulted in ≥ 75% agreement.

**RECOMMENDATIONS:**

Human papillomavirus (HPV) DNA testing is recommended in all resource settings; visual inspection with acetic acid may be used in basic settings. Recommended age ranges and frequencies vary by the following setting: maximal: age 25-65 years, every 5 years; enhanced: age 30-65 years, if two consecutive negative tests at 5-year intervals, then every 10 years; limited: age 30-49 years, every 10 years; basic: age 30-49 years, one to three times per lifetime. For basic settings, visual assessment is used to determine treatment eligibility; in other settings, genotyping with cytology or cytology alone is used to determine treatment. For basic settings, treatment is recommended if abnormal triage results are obtained; in other settings, abnormal triage results followed by colposcopy is recommended. For basic settings, treatment options are thermal ablation or loop electrosurgical excision procedure; for other settings, loop electrosurgical excision procedure or ablation is recommended; with a 12-month follow-up in all settings. Women who are HIV-positive should be screened with HPV testing after diagnosis, twice as many times per lifetime as the general population. Screening is recommended at 6 weeks postpartum in basic settings; in other settings, screening is recommended at 6 months. In basic settings without mass screening, infrastructure for HPV testing, diagnosis, and treatment should be developed.

Additional information is available at www.asco.org/resource-stratified-guidelines.

## INTRODUCTION

The purpose of this guideline is to provide updated expert guidance on the secondary prevention of cervical cancer to clinicians, public health authorities, policymakers, and laypersons in all resource settings. The target population is women in the general population at risk for developing cervical cancer (specific target age depends on the resource level).

There are large disparities regionally and globally in incidence of and mortality resulting from cervical cancer, in part because of disparities in the provision of mass screening and primary prevention. Different regions of the world, both among and within countries, even within districts, differ with respect to access to prevention and, also, treatment.

In addition, marginalized populations within the United States face barriers to cervical cancer screening. Black, Asian, Pacific Islander, Native Hawaiians/other Pacific Islanders, American Indians or Alaskan Natives, and Hispanics are less likely to receive screening compared with White people to undergo cervical cancer screening.^1^ Sexual and gender minority people, particularly sexual and gender minority people of color, are also less likely to undergo cervical cancer screening given a myriad of barriers to primary care including stigma and structural discrimination.^2-5^

American Society of Clinical Oncology (ASCO) published its first guideline on secondary prevention of cervical cancer in 2016.^6^ In 2021 and 2019, the WHO published updated guidelines on screening and treatment and the use of thermal ablation, respectively, for eligible women in all settings; this guideline reinforces those recommendations; in addition, updated US guidelines from American Cancer Society (ACS), American Society for Colposcopy and Cervical Pathology (ASCCP), and US Preventive Task Force were published. The screening modalities addressed by these five guidelines, as well as in this guideline, include cytology (also known as Pap smear), visual inspection (eg, visual inspection with acetic acid [VIA]), and human papillomavirus (HPV) DNA testing (screening); for evaluation of positive results, the WHO guidelines include colposcopy, and for treatment, excisional and ablative treatments. The screening tests are sometimes used and have been studied alone or in combination. The guidelines that ASCO reviewed are described in the Data Supplement (Appendix Table A1). (This ASCO guideline also addresses self-collection and emerging screening technologies.)

**FIG 1:**
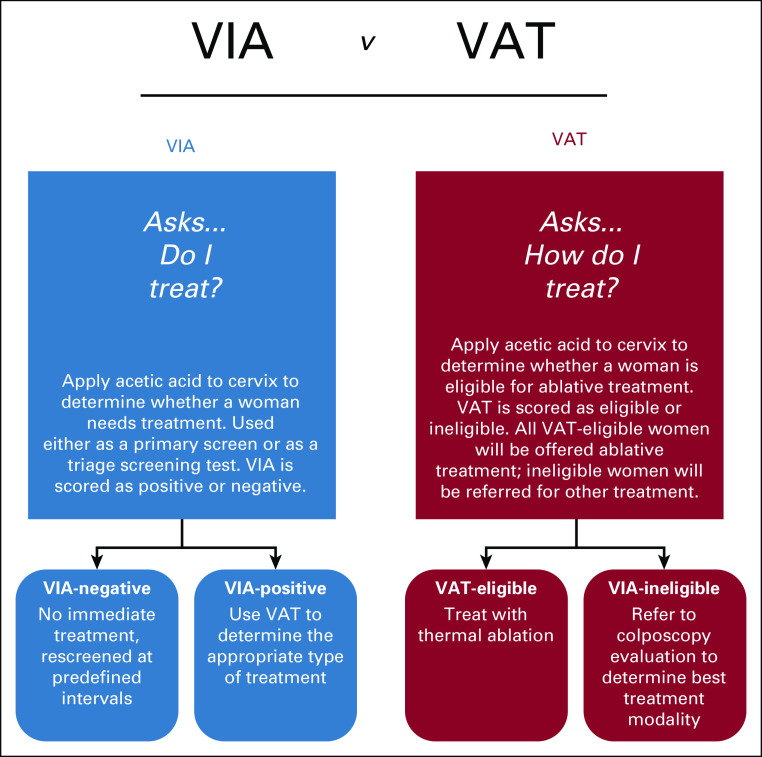
Use of VIA versus use of VAT. VAT, visual assessment for treatment; VIA, visual inspection with acetic acid.

**FIG 2:**
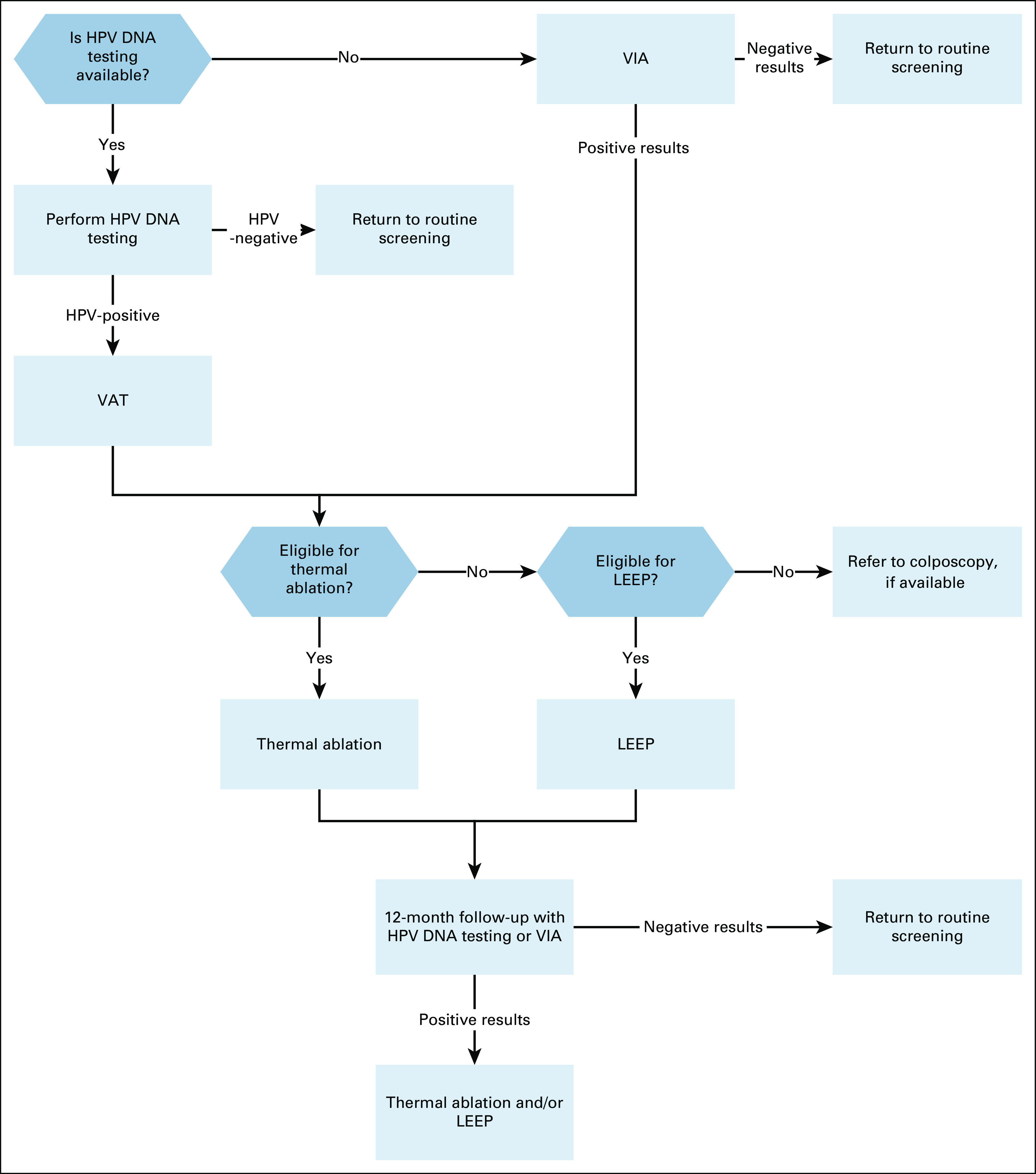
Secondary prevention of cervical cancer for women age 30-49 in basic‐resource settings. HPV, human papillomavirus; LEEP, loop electrosurgical excision procedure; VAT, visual assessment for treatment; VIA, visual inspection with acetic acid.

**FIG 3:**
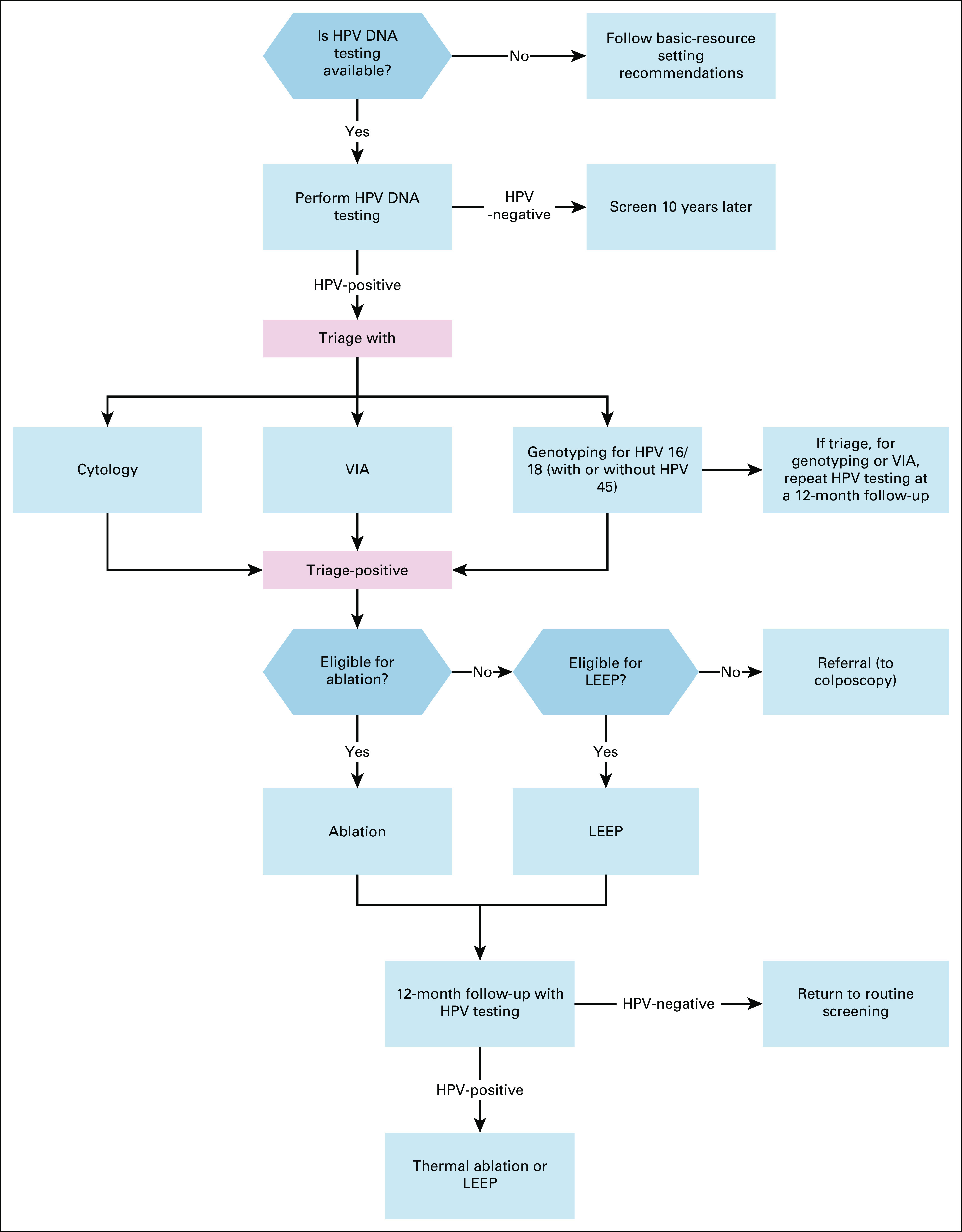
Secondary prevention of cervical cancer for women age 30-49 in limited‐resource settings. HPV, human papillomavirus; LEEP, loop electrosurgical excision procedure; VIA, visual inspection with acetic acid.

**FIG 4:**
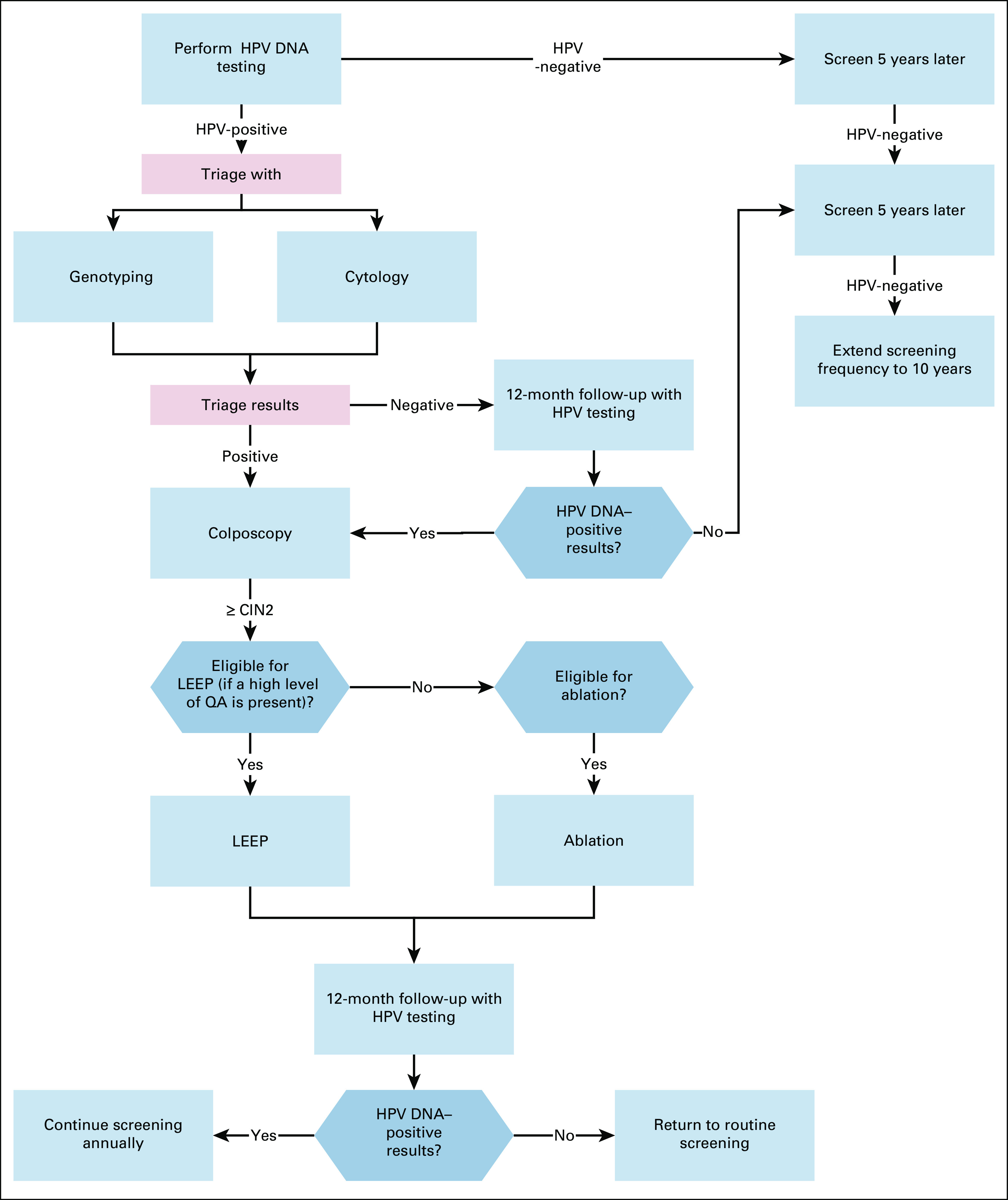
Secondary prevention of cervical cancer for women age 30-65 in enhanced‐resource settings. HPV, human papillomavirus; LEEP, loop electrosurgical excision procedure; QA, quality assurance.

**FIG 5:**
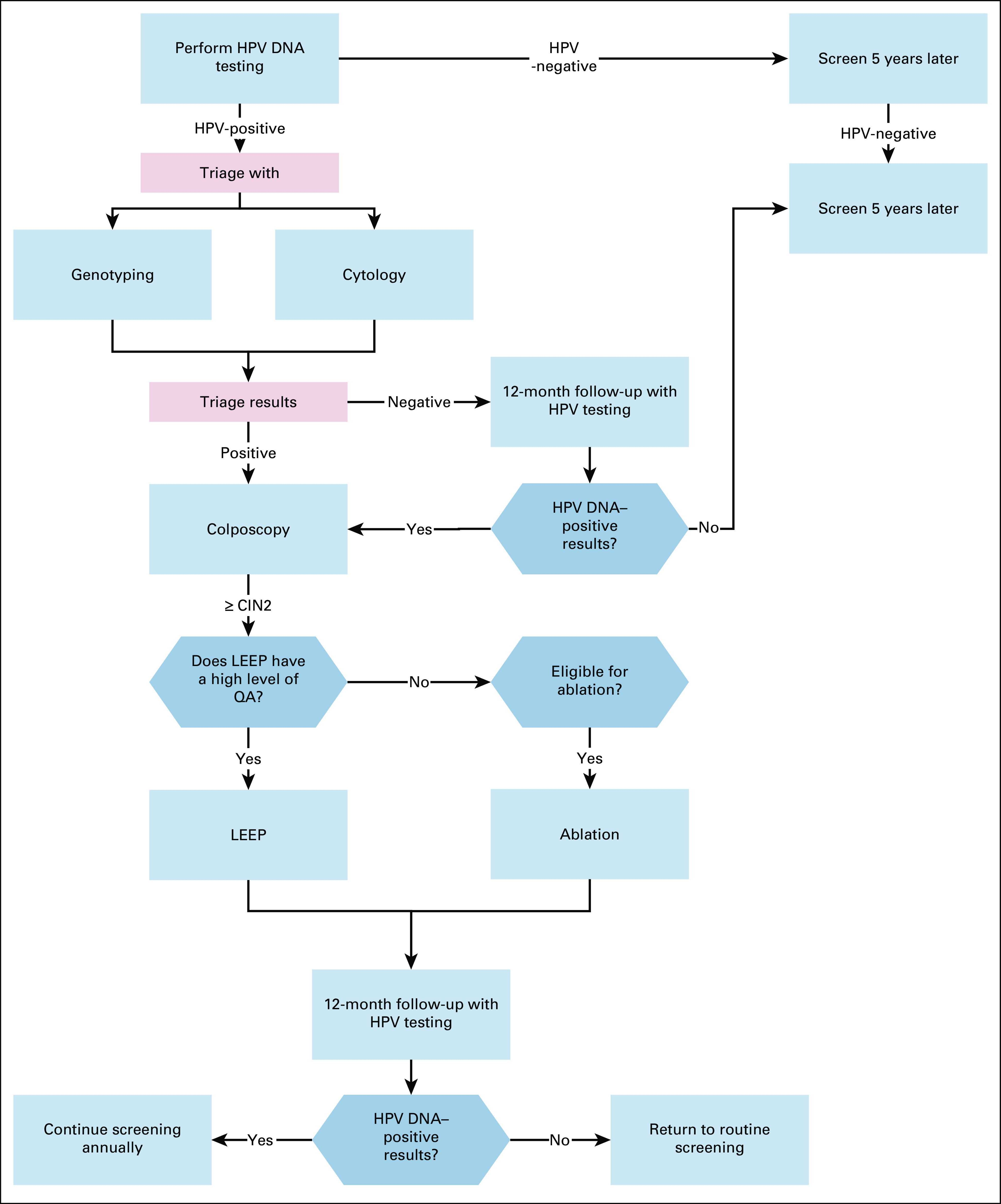
Secondary prevention of cervical cancer for women age 30-65 in maximal‐resource settings. HPV, human papillomavirus; LEEP, loop electrosurgical excision procedure; QA, quality assurance.

ASCO has established a process for resource-stratified guidelines, which includes mixed methods of guideline development, adaptation of the clinical practice guidelines of other organizations, and formal expert consensus. This article summarizes the results of that process and presents updated resource-stratified recommendations, which are based, in part, on formal consensus and adaptation from existing guidelines on the screening, triage of screening results, and treatment of women with cervical cancer precursor lesions (the Results section and Appendix Table A1 list these guidelines).

Although we refer to the sex of cervical cancer screening eligible people as women throughout this guideline document, all people with cervixes are eligible for cervical screening and the guidelines apply uniformly to all such people. This mirrors the statement of WHO in its screen and treat guideline (p. 23), which states, in part, that WHO recommendations “recognize that most of the available evidence on cervical cancer is based on study populations of cisgender women” and “sexual and reproductive health service providers and cervical cancer prevention services must consider the needs of – and provide equal care to – all individuals independently of gender identity or its expression.”^7^

ASCO uses an evidence-based approach to inform guideline recommendations. In developing resource-stratified guidelines, ASCO has adopted its framework from the four-tier approach (basic, limited, enhanced, and maximal; Table 1) developed by the Breast Health Global Initiative and made modifications to that framework on the basis of the Disease Control Priorities 3.^8,9^ Separate ASCO resource–stratified guidelines provide guidance on the treatment of women with invasive cervical cancer^10,11^ and primary prevention.^12^

**THE BOTTOM LINE**

**Secondary Prevention of Cervical Cancer: ASCO Resource–Stratified Guideline Update**

**Guideline Question**

What are the optimal method(s) for cervical cancer screening and the management of women with abnormal screening results for each resource level (ie, basic, limited, enhanced, and maximal)?

**Target Population**

Women who are asymptomatic for human papillomavirus (HPV) infection.

**Target Audience**

Public health authorities, cancer control professionals, policymakers, obstetricians/gynecologists, primary care providers, lay public.

**Methods**

A multinational, multidisciplinary Expert Panel was convened to develop clinical practice guideline recommendations on the basis of a systematic review of existing guidelines and an expert consensus process.

**Author's note:** ASCO's view is that health care providers and health care system decision makers should be guided by the recommendations for the highest stratum of resources available in their setting. The guidelines are intended to complement, but not replace local guidelines.

**Key Recommendations**

**Primary screening.**

- HPV DNA testing is recommended in all resource settings, either self- or clinician-collected.
- The recommended age ranges (years) and frequencies in each setting are as follows:
  - maximal, age 25-65 years every 5 years
  - enhanced, age 30-65 years, if two consecutive negative tests at 5-year intervals, then 10 years
  - limited, age 30-49 years every 10 years
  - basic, not more than every 10 years (with a plan to transition to not less than every 5 years).
- VIA may be used in basic settings and should move to population-based screening with HPV testing at the earliest opportunity.

**Exiting screening.**

- Maximal and enhanced: age 65 years with consistently negative results during the past ≥ 15 years.
- Limited and basic: age 49 years, resource-dependent, see specific recommendations.

**Triage.**

- In basic settings, when a molecular (HPV) test is used for screening and has a positive result, visual assessment for treatment may be used to determine whether the woman should be treated with thermal ablation or loop electrosurgical excision procedure (LEEP).
- For other settings, HPV genotyping with cytology or cytology alone may be used.

**After triage (if a triage test was performed).**

- Women with negative triage results should receive a follow-up HPV test in 12 months (if primary screening was positive and then triage results were negative, then follow-up at 12 months).
- In limited settings, women with abnormal results from triage should receive colposcopy, if available, or treatment, if it is not.
- In maximal and enhanced settings, women with abnormal and/or positive results from triage should receive colposcopy.

**Treatment of women with precursor lesions.**

- In basic and limited settings, treatment options are thermal ablation or LEEP, if a high level of quality assurance is recommended (for LEEP).
- In enhanced and maximal settings, LEEP (if there is a high level of quality assurance) or, where LEEP is contradicted, ablative treatments may be offered.
- A 12-month post-treatment follow-up including HPV testing is recommended for all settings.

**Special populations.**

- Women who are HIV-positive or immunosuppressed for other reasons should be screened with HPV as soon as diagnosed, twice as many times in a lifetime as the general population.
- The management of abnormal results for screening for women with HIV and positive results of triage is the same as in the general population.
- Women who are postpartum should be offered primary screening 6 weeks postpartum in basic settings and at 6 months in other settings.
- Screening may be discontinued in women who have received a total hysterectomy for benign causes with no history of cervical dysplasia or HPV. Women who have received a subtotal hysterectomy (with an intact cervix) should continue receiving routine screening.

**Qualifying statement.**

In basic settings without current mass screening, infrastructure for HPV testing, diagnosis, and treatment should be developed.

Complete recommendations are given in Appendix Table A2 and Figures 2-5.

**Additional Resources**

Definitions for the quality of the evidence and strength of recommendation ratings are available in Appendix Table A3. More information, including a supplement with additional evidence tables, slide sets, and clinical tools and resources, is available at https://www.asco.org/practice-patients/guidelines/resource-stratified. Patient information is available at www.cancer.net.

**ASCO believes that cancer and cancer prevention clinical trials are vital to inform medical decisions and improve cancer care and that all patients should have the opportunity to participate.**

**TABLE 1:**
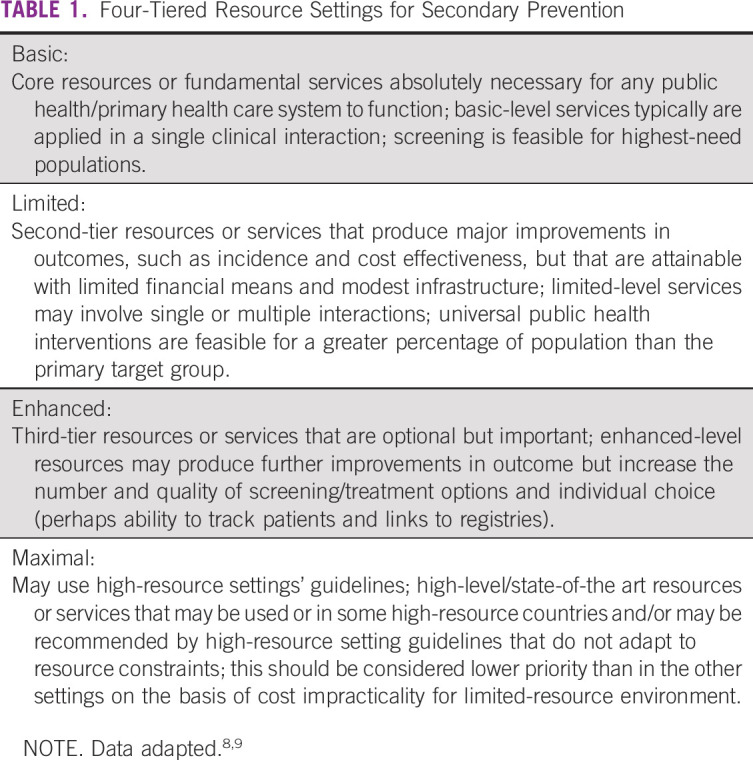
Four-Tiered Resource Settings for Secondary Prevention

## GUIDELINE QUESTIONS

This clinical practice guideline addresses four overarching clinical questions: (1) What are the best method(s) for screening for each resource stratum? (2) What is the best triage and/or management strategy for women with positive results or other abnormal (eg, discordant HPV and/or cytology) results? (3) What are the best management strategies for women with precursors of cervical cancer? (4) What screening strategy should be recommended for women who have received HPV vaccination?

In addition, the guideline addresses screening and management strategies for defined special populations.

## METHODS

These recommendations were developed by an ASCO Expert Panel with multinational and multidisciplinary representation, which included a patient representative and an ASCO guidelines staff member with health research methodology expertise (Appendix Table A4). The Expert Panel met via webinar and corresponded through e-mail. On the basis of the consideration of the evidence, the authors were asked to contribute to the development of the guideline, provide critical review, and finalize the guideline recommendations. The guideline recommendations were sent for Formal Consensus review and for an open comment period of 2 weeks, allowing the public to review and comment on the recommendations after submitting a confidentiality agreement. These comments were taken into consideration while finalizing the recommendations. Members of the Expert Panel were responsible for reviewing and approving the penultimate version of the guideline, which was then circulated for external review and submitted to a peer-reviewed journal for editorial review and consideration for publication. This guideline was partially informed by ASCO's modified Delphi Formal Expert Consensus methodology, during which the Expert Panel was supplemented by additional experts recruited to rate their agreement with the drafted recommendations. The entire membership of experts is referred to as the Consensus Panel (Appendix Table A5 provides a list of members). Eleven experts (plus eight who were on the Expert Panel) participated. All ASCO guidelines are ultimately reviewed and approved by the Expert Panel and the ASCO Evidence–Based Medicine Committee before publication. All funding for the administration of the project was provided by ASCO.

The guideline development process was also informed by the ADAPTE methodology^13^; this and consensus processes were used together as an alternative to de novo recommendation development. Adaptation of guidelines is considered by ASCO in selected circumstances, when one or more quality guidelines from other organizations already exist on the same topic. The objective of the ADAPTE process is to take advantage of existing guidelines to enhance efficient production, reduce duplication, and promote the local uptake of quality guideline recommendations.

The ASCO adaptation and formal expert consensus processes begin with a literature search to identify literature including candidate guidelines for adaptation. An electronic search was conducted to identify any updates to guidelines included in the 2016 guidelines. The panel used existing guidelines, some literature suggested by panel members, and clinical experience as guides. The guideline recommendations were crafted, in part, using the Guidelines Into Decision Support methodology and accompanying BRIDGE-Wiz software.^14^

Adapted guideline manuscripts are reviewed and approved by the Evidence-Based Medicine Committee. The review includes two parts: methodologic review and content review. The former was completed by two ASCO staff members (Data Supplement), and the latter by members of the Expert Panel convened by ASCO.

The ASCO Expert Panel and guidelines staff will work with cochairs to keep abreast of any substantive updates to the guideline. On the basis of the formal review of the emerging literature, ASCO will determine the need to update. The ASCO Guidelines Methodology Manual (available at www.asco.org/guideline-methodology) provides additional information about the guideline update process. This is the most recent information as of the publication date.

### Guideline Disclaimer

The Clinical Practice Guidelines and other guidance published herein are provided by the ASCO to assist providers in clinical decision making. The information herein should not be relied upon as being complete or accurate, nor should it be considered as inclusive of all proper treatments or methods of care or as a statement of the standard of care. With the rapid development of scientific knowledge, new evidence may emerge between the time information is developed and when it is published or read. The information is not continually updated and may not reflect the most recent evidence. The information addresses only the topics specifically identified therein and is not applicable to other interventions, diseases, or stages of diseases. This information does not mandate any particular course of medical care. Further, the information is not intended to substitute for the independent professional judgment of the treating provider, as the information does not account for individual variation among patients. Recommendations specify the level of confidence that the recommendation reflects the net effect of a given course of action. The use of words like “must,” “must not,” “should,” and “should not” indicates that a course of action is recommended or not recommended for either most or many patients, but there is latitude for the treating physician to select other courses of action in individual cases. In all cases, the selected course of action should be considered by the treating provider in the context of treating the individual patient. Use of the information is voluntary. ASCO does not endorse third party drugs, devices, services, or therapies used to diagnose, treat, monitor, manage, or alleviate health conditions. Any use of a brand or trade name is for identification purposes only. ASCO provides this information on an “as is” basis and makes no warranty, express or implied, regarding the information. ASCO specifically disclaims any warranties of merchantability or fitness for a particular use or purpose. ASCO assumes no responsibility for any injury or damage to persons or property arising out of or related to any use of this information, or for any errors or omissions.

### Guideline and Conflict of Interest

The Expert Panel was assembled in accordance with ASCO's Conflict of Interest Policy Implementation for Clinical Practice Guidelines (“Policy,” found at https://www.asco.org/guideline-methodology). All members of the Expert Panel completed ASCO's disclosure form, which requires disclosure of financial and other interests, including relationships with commercial entities that are reasonably likely to experience direct regulatory or commercial impact as a result of promulgation of the guideline. Categories for disclosure include employment; leadership; stock or other ownership; honoraria, consulting or advisory role; speaker's bureau; research funding; patents, royalties, other intellectual property; expert testimony; travel, accommodations, expenses; and other relationships. In accordance with the Policy, the majority of the members of the Expert Panel did not disclose any relationships constituting a conflict under the Policy.

### Note

Where authors are identified as personnel of the International Agency for Research on Cancer and/or WHO, the authors alone are responsible for the views expressed in this article and they do not necessarily represent the decisions, policy, or views of the International Agency for Research on Cancer or WHO.

## RESULTS

### Characteristics of Studies Identified in the Literature Search

As part of the systematic literature review, updates of previous non-ASCO guidelines that ASCO adapted in 2016 were reviewed. On the basis of these reviews of the results, the Expert Panel selected five high-quality guidelines from ACS,^15^ ASCCP,^16^ and US Preventive Task Force^17^ and two from WHO for adaptation.^7,18^ In addition, the IARC perspective on cervical cancer screening informed the context of the guideline update development.^19^

The identified guidelines were published between 2018 and 2021. Five were all or in part systematic review (SR)–based guidelines.^20-26^ All but the WHO guidelines were developed for maximal resource-level settings; the WHO guideline has the largest global constituency. Cost-effectiveness analyses (CEAs) were also reviewed. Because of lack of evidence for some clinical questions, formal expert consensus was used.

An overview of and information on these guidelines' clinical questions, target populations, development methodology, and key evidence is presented in the Data Supplement. The evidence and guidelines supporting unchanged recommendations are reviewed in the previous guideline publication.^6^

## RECOMMENDATIONS (ONLY CHANGES ARE DISCUSSED BELOW)

### Primary Screening

#### Recommendations 1.1, 2.1, 3.1, and 4.1 (all four settings).

**Recommendation 1.1 (Maximal):** In maximal-resource settings, cervical cancer screening with HPV DNA testing should be offered every 5 years from ages 25 to 65 years (either self- or clinician-collected). On an individual basis, women may elect to receive screening until 70 years of age. (Type: evidence-based for test, interval, and age [25-65 years]; Type: formal consensus-based [until age 70 years]; Evidence quality: high; Strength of recommendation: strong.)

**Recommendation 2.1 (Enhanced):** In enhanced-resource settings, cervical cancer screening with HPV DNA testing should be offered to women 30-65 years of age, every 5 years (ie, second screen 5 years from the first) (either self- or clinician-collected). (Type: evidence-based; Evidence quality: high; Strength of recommendation: strong.)

**Recommendation 3.1 (Limited):** In limited settings, cervical cancer screening with HPV DNA testing should be offered to women 30-49 years of age every 10 years, corresponding to two to three times per lifetime (either self- or clinician-collected). (Type: evidence-based [age range]; Type: formal consensus-based [interval]; Evidence quality: intermediate; Strength of recommendation: moderate.)

**Recommendation 4.1 (Basic):** Health systems in basic settings should move to population-based screening with HPV testing at the earliest opportunity (either self- or clinician-collected). If HPV DNA testing for cervical cancer screening is not available, then VIA should be offered with the goal of developing health systems. Screening should be offered to women 30-49 years of age, at least every 10 years (increasing the frequency to every 5 years resources permitting). (Type: evidence-based; Evidence quality: intermediate; Strength of recommendation: strong.)

##### Discussion

All the primary screening recommendations now include self-collection as an option for samples to test. This is consistent with WHO guidance, discussed in the IARC Handbook,^27^ and the addition is a change from the previous ASCO guideline. Self-collection may be more acceptable to some people, including transgender people, less resource-intensive than clinician-collected samples, and its accuracy is like clinician-collected samples.^28^ WHO based its recommendation on a meta-analysis of HPV testing on self-collection versus clinician-collected samples, Arbyn et al^29^ originally performed in 2018 and updated in 2020, which showed similar specificity and sensitivity on polymerase chain reaction–based HPV DNA testing. In addition, it may be more cost-effective. Self-collection is highly sensitive and feasible; regions should start pilot projects at a minimum.

ASCO continues to recommend HPV DNA screening over cytology or cotesting (ie, HPV DNA testing plus cytology) in all settings and 5-year frequency in maximal and enhanced settings, with encouragement that limited and basic settings move toward this frequency.

##### Cost-effectiveness analyses

The 2016 guideline reviewed several CEAs—this section presents data from CEAs conducted since then.

*Maximal-resource settings*: A cost-effectiveness study modeling screening of unvaccinated women in Sweden found that primary HPV testing was more cost-effective than alternative strategies.^30^ A cost-effectiveness study on the basis of a randomized clinical trial (RCT) in Canada found that primary HPV testing was less costly and more effective at detecting precursor lesions than cytology-based screening. A modeling study from Norway found that self-collected HPV testing may be more cost-effective than clinician-collected HPV testing^31^ screening programs, and other studies project that HPV self-collection offered to underscreened women may increase health benefits and be cost-effective.^32,33^

*Limited-resource settings*: Several CEAs in middle-income countries (ie, Iran, El Salvador, and China) have also examined primary HPV screening intervals of 5 years and found these to be an efficient use of resources.^34-36^ In settings where screening coverage is low, it is generally more cost-effective to expand coverage of unscreened women rather than increasing the number of lifetime screenings among women who already have access.^37^

Depending on the delivery model and uptake among underscreened women, HPV self-collection may be a cost-effective strategy to expand coverage in low-resource settings and among underscreened people, including transgender people, in higher-resource settings.^3,33,38-42^

#### Recommendation 4.1—screening in basic settings.

Health systems in basic settings should move to population-based screening with HPV testing at the earliest opportunity (either self- or clinician-collected). If HPV DNA testing for cervical cancer screening is not available, then VIA should be offered with the goal of developing health systems. Screening should be offered to women 30-49 years of age, not more than every 10 years (with a plan to transition to not less than every 5 years). This section specifically regards the portion that says “Health systems in basic settings should move to population-based screening with HPV testing at the earliest opportunity (either self- or clinician-collected). If HPV DNA testing for cervical cancer screening is not available, then VIA should be offered with the goal of developing health systems.”

##### Discussion

These updated recommendations are based on the WHO guideline and formal consensus. WHO recommends “using HPV DNA detection as the primary screening test rather than VIA or cytology” (source: WHO screen and treat,^7^ p. xi, recommendation 1 and recommendation 21 [latter for those with HIV]). WHO considered the balance of benefits and risks when comparing VIA and HPV DNA testing and based the recommendation on RCTs and modeling.

In basic settings, where there is no mass screening and no culture of screening, VIA may be used, with the goal of moving to population-based screening with HPV testing at the earliest opportunity.

In addition, the recommendation continues to state that in limited settings, the recommended age range is 30-49 years. When more resources become available, policymakers may consider extending the upper age range.

##### Cost-effectiveness analyses

*Basic resource settings*: In basic resource settings, a SR found that the relative cost-effectiveness of primary HPV testing and VIA depends on screening program characteristics, including HPV test cost, adherence to recommended follow-up, and performance of VIA.^43,44^

### Triage

#### Maximal and enhanced.

There are no changes in the maximal and enhanced setting recommendations for triage.

**Recommendation 3.2 (Limited):** If the results of the HPV DNA test are positive, clinicians should then perform triage with reflex cytology (quality assured) and/or HPV genotyping for HPV 16/18 (with or without HPV 45) or with VIA. If institutions are currently using reflex cytology, they should transition from cytology to HPV genotyping. (For cytology and genotyping; Type: evidence-based; Evidence quality: high; Strength of recommendation: strong; for VIA; Type: formal consensus-based; Evidence quality: low; Strength of recommendation: weak.)

Qualifying statement: In limited settings, the preference is to do direct treatment, with triage using partial genotyping.

##### Discussion

In basic settings, visual assessment for treatment (VAT) after positive HPV DNA results is recommended to determine the appropriate type of treatment (Fig 1). Regarding the portion of Recommendation 3.2 (Limited) with the inclusion of the statement that if institutions are currently using reflex cytology, they should transition from cytology to HPV genotyping.

The Panel recommends that in basic settings where a molecular (HPV) test is used as the primary screening test, and further triage testing is not feasible, all positive results are referred for VAT to determine whether ablation or excisional procedures should be used for treatment. When programs use self-collection, partial genotyping may be more possible because of its lower cost than that of cytology. Partial genotyping is preferred because it is less resource-intensive, and if the patient does not have a high-risk variant of HPV, they may not need treatment. The Panel added a qualifying statement preferring provision of direct treatment in limited settings to reduce the number of visits that a person in the screening algorithm must make. Direct treatment of everyone who has positive primary screening results, aka screen-and-treat, can reduce the possibility of losing patients to follow-up.

If direct treatment is not feasible or acceptable, the options could be either partial genotyping or treating only those who are HPV 16/18–positive or VIA-based triage, as enumerated in the 2019 ASCCP guidelines.^16^ Although even large programs might not have that option, over time, most programs will transition to self-sampling with partial genotyping. The recommendations are based on the trade-off between feasibility and desirability of high-quality impact, noting that different health systems and countries will have to weigh clinical performance with the ability to perform certain treatments and tests.

##### Cost-effectiveness analyses

The cost-effectiveness of HPV screen-and-treat versus HPV screen-triage-treat algorithms (with triage strategies including HPV 16/18 genotyping, VIA, colposcopy, and cytology) has been evaluated in several studies.^7,44-46^ HPV screen-and-treat has generally been found to be a more cost-effective strategy, because of limited screening opportunities in lower-resource settings and the need to maximize detection of people at high risk of cervical cancer; however, the high burden of HPV in some settings may overwhelm the capacity to provide treatment, making triage testing necessary. Few studies have examined the cost-effectiveness of HPV 16/18 genotyping in limited settings, but it appears to have similar costs and only slightly lower benefits than HPV screen-and-treat.^7^

**Recommendation 3.3 (Limited):** If cytology triage results are abnormal (ie, ≥ atypical squamous cells of undetermined significance), women should be referred to quality-assured colposcopy (the first choice, if available and accessible for women who are ineligible for thermal ablation), during which biopsies of any acetowhite (or suggestive of cancer) areas should be taken, even if the acetowhite lesion might appear insignificant. If colposcopy is not available, then perform VAT. (Type: evidence-based; Evidence quality: intermediate; Strength of recommendation: moderate.)

*Addition of clarification of use of colposcopy in limited*: The Panel added women who are ineligible for thermal ablation to the cytology triage recommendation. If a person has positive results and is eligible for thermal ablation and with a low suspicion of cancer, they can proceed to treatment without colposcopy, in part, to lessen number of recalls. However, if clinicians have a higher suspicion of cancer and patients have factors making them ineligible for ablation, colposcopy is used to rule out cancer. This is in line with the ASCCP guidance and the WHO thermal ablation guideline.

### Treatment

#### Maximal and enhanced.

There are no changes in the maximal and enhanced setting recommendations.

**Recommendation 4.2 (Basic):** If the results of available HPV testing are positive, clinicians should then perform VAT followed by treatment with thermal ablation and/or loop electrosurgical excision procedure, depending on the size and location of the lesion. (Type: formal consensus-based; Evidence quality: low; Strength of recommendation moderate.)

**Recommendation 4.3 (Basic):** If primary screening is VIA and results are positive, then treatment should be offered with thermal ablation and/or loop electrosurgical excision procedure, depending on the size and location of the lesion. (Type: evidence-based; Evidence quality: intermediate; Strength of recommendation: moderate.)

Ablation is now recommended in basic settings (as it was already in limited settings).

##### Discussion

In 2016, the ASCO guideline did not recommend thermal ablation over other treatments for patients with precursor lesions in the basic setting; it was already recommended in limited settings. More evidence has become available since that time. WHO produced a guideline on thermal ablation, published in 2019, comparing ablation with cryotherapy and surgical approaches. For the 2019 version, WHO used evidence from RCTs, SRs, and meta-analyses and data from ongoing trials and assessed the evidence with Grading of Recommendations, Assessment, Development and Evaluations. They found no difference between benefits and harms of cryotherapy and thermal ablation.

Cryotherapy is less feasible, has issues with availability of parts of its process (eg, with supply), and is more costly.^18^ Therefore, ablation is more feasible and at lower cost, and providers may find it more acceptable and usable with less delay than cryotherapy. Therefore, the Expert Panel states a preference for ablation and recommends institutions transition from cryotherapy.

#### Maximal and enhanced.

Overall: There are no changes in the maximal and enhanced setting recommendations, except for self-collection.

##### ASCO recommendations for special populations

Recommendations for populations such as those with HIV have not changed and are consistent with WHO recommendations.

##### ASCO recommendations on the screening strategy for women who have received HPV vaccination

The previous recommendation to continue screening in women who have received HPV vaccination is still in force. Recommendations for women who have received HPV vaccination have not changed, and these populations should continue to receive screening. HPV-based screening at 5-year intervals is recommended for women who have received HPV vaccination until there are further data on the efficacy of the currently available HPV vaccines in preventing all cases of CIN2+.

###### New screening technologies.

The 2016 guideline contained a section on new technologies being investigated for all resource setting levels. Most are still under investigation. Novel technologies—under development or undergoing field tests—for treating precancerous lesions include ablative technologies such as thermocoagulation, portable cryotherapy devices that rely less on gas, therapeutic vaccines, antivirals, and topical applications.^47^

## COST AND POLICY IMPLICATIONS

The secondary prevention of cervical cancer is a cost-effective strategy to reduce the incidence and mortality of cervical cancer. CEAs discussed in this guideline support the use of HPV DNA tests in maximal, enhanced, limited, and basic resource settings. However, there are specific implementation issues regarding providing screening and treatment in limited and basic settings in primary care, outside of research studies.

In addition to cost and policy implications discussed in the 2016 guideline and in this version, other delivery strategies may involve some combination of screening campaigns, mobile clinics, and HPV self-sampling.^48,49^ These strategies can improve screening uptake and linkage to treatment for screen-positive women. Although several novel biomarkers for triage of HPV-positive women remain to be validated, incorporating triage tests that identify women at high risk of cervical precancer may improve the cost-effectiveness of screening if fewer women can be treated while avoiding false-negative screening results. In addition to providing value, accurate triage tests may avert overburdening health care systems in settings with a high prevalence of HPV.

## EXTERNAL REVIEW AND OPEN COMMENT

The draft recommendations were released to the public for open comment from April 12 through April 22, 2022. Response categories of “Agree as written,” “Agree with suggested modifications,” and “Disagree. See comments” were captured for every proposed recommendation with 34 written comments received. A total of 85%-100% of the 34 responses either agreed or agreed with slight modifications to the recommendations, and 0%-15% of the responses disagreed. Expert Panel members reviewed comments from all sources and determined whether to maintain original draft recommendations, revise with minor language changes, or consider major recommendation revisions. All changes were incorporated before Evidence-Based Medicine Committee review and approval.

## GUIDELINE IMPLEMENTATION

ASCO guidelines are developed for implementation across health settings. Barriers to implementation include the need to increase awareness of the guideline recommendations among front-line practitioners and women in general populations and also to provide adequate services in the face of limited resources. Implementation should reflect the latest implementation research. The guideline Bottom Line Box was designed to facilitate the implementation of recommendations. This guideline will be distributed widely, including through many forms of ASCO communications and the ASCO website.

## LIMITATIONS OF RESEARCH

There were limitations in the evidence regarding screening after HPV vaccination and emerging technologies. Future research is suggested in these areas.

## FUTURE DIRECTIONS

In addition to addressing research limitations, future research is needed in other areas, eg, self-collection, biomarkers, needs and preferences of women/people with cervixes, improving screening for transgender people, cost-effective strategies, low-cost technology, and the impact of vaccination on screening.

Addressing policy and health system barriers may include the following:

- Education of medical and public health communities to change practices and incorporate new technologies
- Participation and sponsorship from policymakers
- Partnerships with institutions, regions, and countries with treatment facilities
- Coordinated volume purchasing and procurement of HPV testing
- Improvement of health information systems to have better follow-up and treatment of women with positive screening results
- Quality control
- Monitoring and evaluation
- Assessing the impact of COVID-19 disease

**ASCO believes that cancer and cancer prevention clinical trials are vital to inform medical decisions and improve cancer care and that all patients should have the opportunity to participate.**

## ADDITIONAL RESOURCES

Additional information including a supplement with additional tables and clinical tools and resources is available at www.asco.org/resource-stratified-guidelines. Patient information is available at www.cancer.net.

## GENDER-INCLUSIVE LANGUAGE

ASCO is committed to promoting the health and well-being of individuals regardless of sexual orientation or gender identity.^50^ Transgender and nonbinary people, in particular, may face multiple barriers to oncology care including stigmatization, invisibility, and exclusiveness. One way that exclusiveness or lack of accessibility may be communicated is through gendered language that makes presumptive links between gender and anatomy.^51-54^ With the acknowledgment that ASCO guidelines may affect the language used in clinical and research settings, ASCO is committed to creating gender-inclusive guidelines. For this reason, guideline authors use gender-inclusive language whenever possible throughout the guidelines. In instances in which the guideline draws upon data on the basis of gendered research (eg, studies regarding women with ovarian cancer), the guideline authors describe the characteristics and results of the research as reported.

**RELATED ASCO GUIDELINES**

**Resource-Stratified Guidelines**

- Management and Care of Women With Invasive Cervical Cancer^10,11^ (https://ascopubs.org/doi/full/10.1200/GO.22.00027 and https://ascopubs.org/doi/full/10.1200/JGO.2016.003954)
- Primary Prevention of Cervical Cancer^12^ (https://ascopubs.org/doi/full/10.1200/JGO.2016.008151)

**Nonresource-Stratified Guidelines**

- Patient-Clinician Communication (http://ascopubs.org/doi/10.1200/JCO.2017.75.2311)

## Figures and Tables

**TABLE A1:**
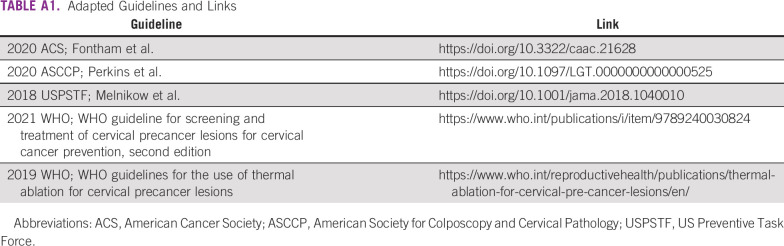
Adapted Guidelines and Links

**TABLE A2:**
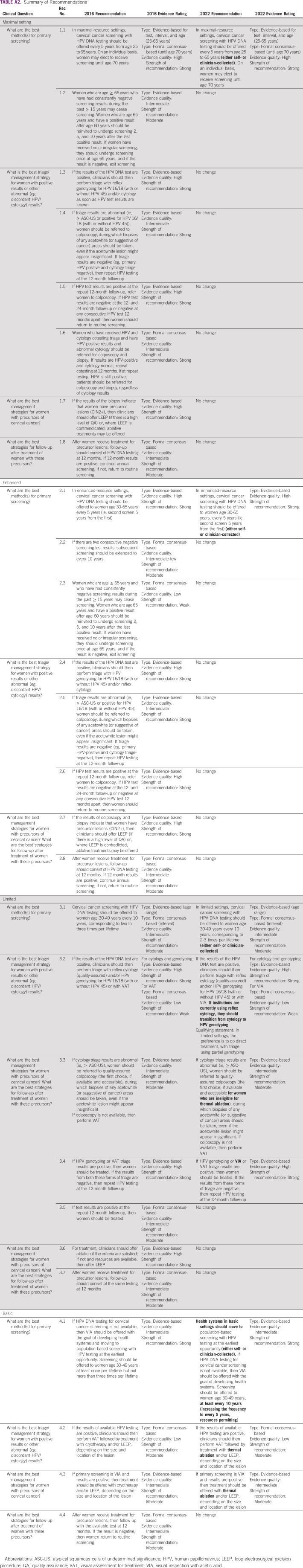
Summary of Recommendations

**TABLE A3:**
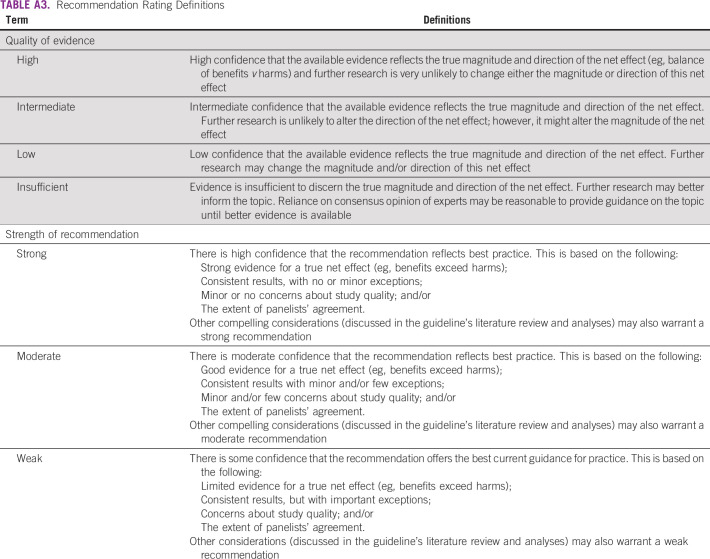
Recommendation Rating Definitions

**TABLE A4:**
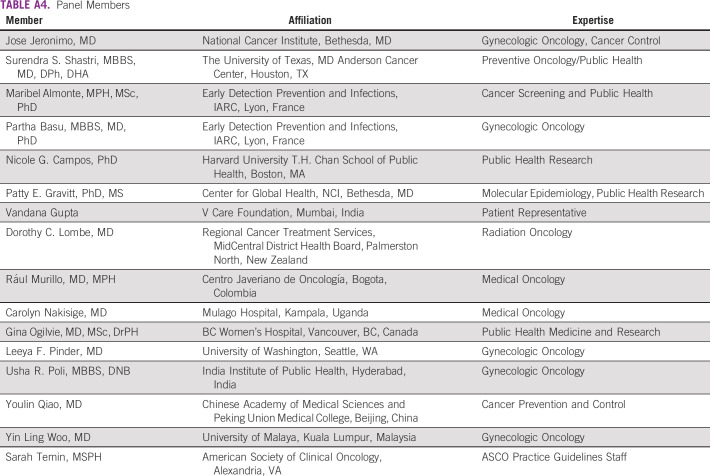
Panel Members

**TABLE A5:**
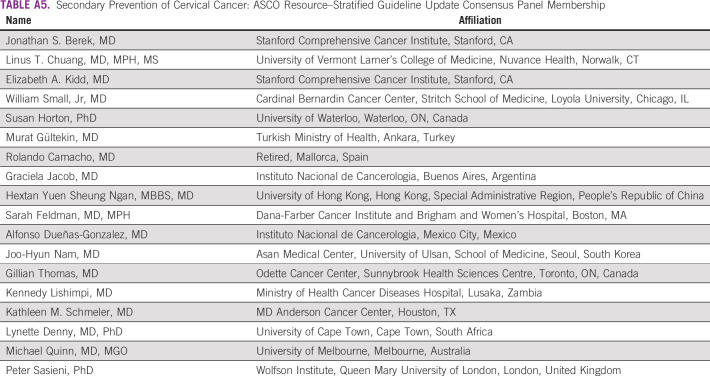
Secondary Prevention of Cervical Cancer: ASCO Resource–Stratified Guideline Update Consensus Panel Membership

## EDITOR’S NOTE

This American Society of Clinical Oncology (ASCO) Clinical Practice Guideline provides recommendations, with comprehensive review and analyses of the relevant literature for each recommendation. Additional information, including a supplement with additional evidence tables, slide sets, clinical tools and resources, and links to patient information at www.cancer.net, is available at www.asco.org/resource-stratified-guidelines.
